# Nomogram for overall survival of Japanese patients with bone-metastatic prostate cancer

**DOI:** 10.1186/s12885-015-1330-x

**Published:** 2015-05-01

**Authors:** Yasuhide Miyoshi, Kazumi Noguchi, Masahiro Yanagisawa, Masataka Taguri, Satoshi Morita, Ichiro Ikeda, Kiyoshi Fujinami, Takeshi Miura, Kazuki Kobayashi, Hiroji Uemura

**Affiliations:** 1 Department of Urology, Yokohama City University Medical Center, Yokohama, Japan; 2 Department of Urology, Yokohama City University School of Medicine, Yokohama, Japan; 3 Department of Biostatistics and Epidemiology, Yokohama City University School of Medicine, Yokohama, Japan; 4 Department of Urology, Yokohama Minami Kyosai Hospital, Yokohama, Japan; 5 Department of Urology, Kanagawa Cancer Center, Yokohama, Japan; 6 Department of Urology, Yokosuka Kyosai Hospital, Yokosuka, Japan

**Keywords:** Prostate cancer, Nomogram, Prognostic tool, Bone metastasis

## Abstract

**Background:**

We analyzed the relationship between prostate cancer outcomes and pretreatment clinical factors and developed a prognostic nomogram of overall survival (OS) of patients with bone metastasis.

**Methods:**

From 1993 to 2011, 463 consecutive patients were treated for bone-metastatic prostate cancer. Data sets from 361 patients were used to develop a nomogram (training data), and data sets of 102 patients were used for validation of the nomogram (validation data). Using the external validation data set, the nomogram was assessed for discriminatory ability, and the predictions were assessed for calibration accuracy by plotting actual survival against predicted risk.

**Results:**

Of the 361 patients in the training data set, 205 (56.8%) patients died, 169 (46.8%) deaths of which were due to prostate cancer. The median follow-up period was 55.2 months. In the multivariate analysis, patient age, serum prostate-specific antigen level, clinical T stage, extent of disease on bone scan, and biopsy Gleason sum were independent prognostic factors. We developed a prognostic model comprising these five factors for patients with bone-metastatic prostate cancer. This nomogram can be used to estimate 1-, 3-, and 5-year survival probability. External validation of this model using 102 validation data sets showed reasonable accuracy (concordance index, 0.719).

**Conclusion:**

Our pretreatment prognostic nomogram might be useful for Japanese patients with bone-metastatic prostate cancer.

## Background

Prostate cancer is the most common noncutaneous cancer, and the second most frequent cause of death from cancer among men in the US. In Japan, 10,722 patients (17.4 per 100,000) died of prostate cancer in 2010, making this disease the sixth leading cause of cancer death [1]. The incidence of prostate cancer is lower in Japan than in the US and other Western countries; however, its incidence has been gradually increasing in Japan in recent years [1]. Huggins and Hodges [2] reported the efficacy of androgen deprivation therapy for advanced prostate cancer in 1941. Although 80–90% of prostate cancers with metastasis respond to initial androgen ablation therapy, most patients will ultimately develop progressive disease. Although some patients can obtain benefit from second-line hormone therapy, anti-androgen withdrawal therapy, new hormonal therapy such as enzalutamide and abiraterone, or chemotherapy, most patients finally develop castration-resistant prostate cancer (CRPC) [3,4]. Patients with CRPC show progression of systemic symptoms and local complications. One report showed that the median survival duration among patients with advanced prostate cancer was 29 to 34 months from initial treatment [5], and another study reported a 5-year survival rate of 20–30% [6]. Because these reports showed a wide range of survival probability, more accurate information on patient characteristics related to survival is needed.

In the US and Europe, some new effective agents for CRPC have been approved, such as docetaxel, cabazitaxel, sipuleucel-T, abiraterone, and enzalutamide [7-11]. Unfortunately, treatment for CRPC was still very limited in Japan until 2013 (cabazitaxel, abiraterone, and enzalutamide were approved in 2014), although docetaxel has been approved [12]. Survival of patients with CRPC is predicted to improve with the use of these drugs. Several groups have reported prognostic models for survival of patients with progressive disease. Almost all reports were of a prognostic nomogram for patients with CRPC; there are few reports about a prognostic nomogram for patients with metastatic prostate cancer before treatment. A large study on the prognosis of patients with pre-hormonal therapy prostate cancer was reported in Japan and the US [13]; however, the endpoint was not survival, but recurrence. Our interest is in the development of an overall survival (OS) prognostic model for hormone-naïve metastatic prostate cancer. Accurate prediction models for prostate cancer survival would be valuable for patient counseling. We analyzed the relationship between prostate cancer outcomes and pretreatment clinical factors and developed a prognostic nomogram for OS of patients with bone metastasis. Our pretreatment prognostic nomogram might be useful for Japanese patients with bone-metastatic prostate cancer.

## Methods

### Patients and treatments

From 1993 to 2011, 463 consecutive patients with bone-metastatic prostate cancer were treated at Yokohama City University Hospital and associated hospitals. All patients already had metastasis at the time of diagnosis, and none of the patients had been previously treated. The data sets of 361 patients from Yokohama City Medical Center, Yokohama City University Hospital, Kanagawa Cancer Center, Minami Kyosai Hospital, Chigasaki Hospital, and Fujisawa Municipal Hospital were used to develop a nomogram (training data), and the data sets of 102 patients from Kawasaki Ida Hospital, International Goodwill Hospital, and Yokosuka Kyosai Hospital were used for validation of the nomogram (validation data).

All patients had adenocarcinoma of the prostate, confirmed histologically, with bone metastasis (any T, any N, M1b). The 2009 TNM clinical staging system and 2005 International Society of Urologic Pathology Gleason grading system were used. In all patients, clinical stage was evaluated by chest and body computed tomography and bone scans. Based on the number or extent of metastases, the scans were divided into the following five grades according to the extent of disease on bone scan (EOD) [14]: 0, normal or abnormal due to benign bone disease; 1, fewer than 6 bony metastases, each of which is less than 50% of the size of a vertebral body (1 lesion about the size of a vertebral body was counted as 2 lesions); 2, from 6 to 20 bone metastases, sized as described above; 3, more than 20 metastases but fewer than seen in a “superscan”; and 4, “superscan” or its equivalent, *i.e.,* more than 75% of the ribs, vertebrae, and pelvic bones.

Docetaxel therapy was not included as a covariate because the nomogram was used as a tool to predict pretreatment survival.

Each hospital used the same treatment protocol. All patients were initially treated with androgen deprivation therapy (medical or surgical castration with or without anti-androgen). After failed initial androgen ablation therapy, almost all patients were subsequently underwent substitution treatment comprising anti-androgen therapy, anti-androgen withdrawal therapy, and/or oral low-dose steroid therapy. Some patients received a bisphosphonate and cytotoxic therapy such as docetaxel or estramustine after development of CRPC. In the terminal state, palliative therapy and pain control with morphine, palliative external beam radiation, and strontium were used as appropriate.

### Statistical analysis

The nomogram was developed using a Cox proportional hazards regression model with stepwise regression analysis. The predictive variables for the nomogram were patient age at initial treatment, serum prostate-specific antigen (PSA) level before treatment, clinical T stage, EOD to classify the extent of bone metastasis, and the biopsy Gleason sum. Relative risks and 95% confidence intervals were derived. The nomogram for OS was developed from the results of the Cox proportional hazards model.

Calibration of the nomogram predictions was evaluated by comparing the predicted probability at 5 years with the Kaplan–Meier survival probability using the training data (internal calibration). We also evaluated the calibration by comparing the predicted probability at 3 years with the Kaplan–Meier survival probability using the external validation data (external calibration). Using the validation data set, the nomogram was assessed for discriminatory ability by quantifying the concordance index (c-index), and the predictions were assessed for calibration accuracy by plotting actual survival against predicted risk. The Kaplan–Meier product-limit estimator was used to estimate the survival distribution. The chi-squared test and Mann–Whitney U test were used to assess the difference in baseline factors between the training data set and the validation data set. The log-rank test was used to analyze differences in survival probability between the training data set and the validation data set. All tests were two-sided, and the significance level was fixed at alpha = 0.05. All analyses were conducted with IBM SPSS Statistics for Windows, ver. 19 (IBM Corp., Armonk, NY) and the R stats package (R Foundation for Statistical Computing, Vienna, Austria). Informed consent was obtained from all patients, and the experimental procedures were conducted in accordance with the ethical standards of the Helsinki Declaration. This study was approved by each of the participating institutions’ review boards Yokohama City University Medical Center, Yokohama City University Hospital, Yokohama Minami Kyosai Hospital, Kanagawa Cancer Center, Yokosuka Kyosai Hospital, Chigasaki Hospital, Kawasaki Ida Hospital, International Goodwill Hospital, and Fujisawa Municipal Hospital).

## Results

### Training data

**Figure 1:**
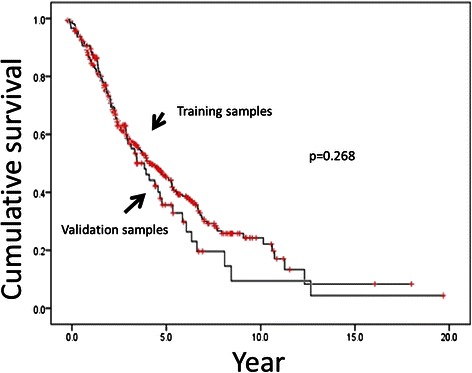
Overall survival in training and validation samples obtained from Kaplan–Meier estimates.

The pretreatment characteristics of the 361 patients included in the training data set are listed in Table 1. Of these patients, 205 (56.8%) died, 169 (46.8%) deaths of which were due to prostate cancer. The median OS was 55.6 months (95%CI: 45.1-66.1), and the cause-specific survival duration was 68.0 months (95%CI: 53.0-83.0). The OS of the patients included in the training data set is shown in Figure 1. In the training data set, 69 (19.1%) patients received docetaxel for treatment of CRPC.

**Table 1:** **Baseline characteristics of training and validation samples**

Variables	Training sample	Validation sample	p value
No. of patients	361	102	
Age, years (mean, SD)	71.43 (8.68)	70.39 (8.17)	0.309**
PSA, ng/mL (median, IQR)	253.8 (728.3-1349.7)	358.0 (652.0-1597.2)	0.779**
≤T3, T4 (%)	81.1, 18.9	62.8, 37.2	<0.0001*
EOD 1, 2, 3, 4 (%)	40.7, 26.6, 25.5, 7.2	35.3, 27.5, 28.4, 8.8	0.764*
Gleason ≤6, 7, 8-10 (%)	6.4, 18.6, 75.0	3.0, 16.7, 80.3	0.189*
Use of docetaxel (%)	19.1	35.3	<0.0001*
Observation period, years (median, IQR)	3.11 (3.11-4.15)	2.58 (2.58-3.51)	0.027**

*Chi-squared test.

**Mann–Whitney U test.

SD: standard deviation.

IQR: interquartile range.

EOD: extent of disease on bone scan.

### Multivariate analysis

**Figure 2:**
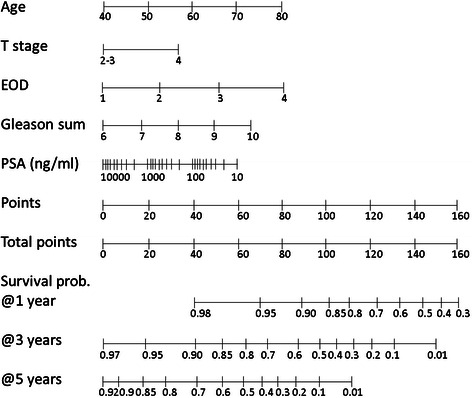
Nomogram of prediction of OS in patients with bone-metastatic prostate cancer.

In the multivariate analysis, patient age at initial treatment, pretreatment serum PSA level, clinical T stage, EOD, and biopsy Gleason sum were independent prognostic factors. Table 2 shows the results of the multivariate analysis, on which the nomogram was based. These five factors were included in the final nomogram. Figure 2 shows a nomogram that can predict the OS of patients with bone-metastatic prostate cancer. This nomogram can be used to estimate the 1-, 3-, and 5-year survival probability. Each scale position has corresponding prognostic points located on the “Points” scale. To determine the points of each factor, a vertical line is drawn from each factor axis to the “Points” axis. The point values for all five predictors are summed to arrive at the “Total points” value.

**Table 2:** **Multivariate model predicting overall survival**

Parameter	p-value	Hazard ratio	HR lower CI	HR upper CI
Age	0.0002	1.035	1.016	1.054
T stage	0.0002	1.882	1.345	2.634
EOD2	0.0221	1.552	1.065	2.260
EOD3	<.0001	2.472	1.664	3.673
EOD4	<.0001	4.042	2.291	7.132
Gleason score	0.0002	1.334	1.144	1.555
Log PSA	0.0023	0.712	0.572	0.886

EOD: extent of disease on bone scan.

GS: Gleason sum.

HR lower CI: hazard ratio, lower 95% confidential interval.

HR upper CI: hazard ratio, upper 95% confidential interval.

### Validation data

The pretreatment characteristics of the 102 patients included in the training data set are listed in Table 1. These data were obtained from three hospitals, including one hospital that performed a docetaxel-related clinical trial. Of these patients, 55 (53.9%) died, 44 (43.1%) deaths of which were due to prostate cancer. The median OS was 48.3 months (95%CI: 36.1-60.5), and the cause-specific survival was 54.9 months (95%CI: 43.8-65.9). The OS for the patients in the validation data set is shown in Figure 1. There was no difference in the OS between the training data set and the validation set (p = 0.268). In the validation data set, 36 (35.3%) patients received docetaxel for treatment of CRPC. The docetaxel-use rate was significantly higher in the validation data set than in the training data set (19.1%) (p < 0.0001). Moreover, there were significant differences in the incidence of clinical T4 disease between the training data and the validation data (p < 0.0001).

We evaluated the discriminatory ability of the nomogram by quantifying the c-index, and the predictions were assessed for calibration accuracy by plotting actual survival against predicted risk using the external validation data set. The c-index of the nomogram was 0.719.

### Internal and external validations

**Figure 3:**
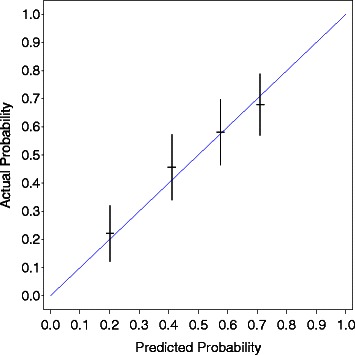
Internal calibration of nomogram of 5-year survival. The predicted survival rate from the nomogram was well correlated with the actual observation. The blue line indicates the ideal reference line where the predicted probabilities match the observed proportions. The dashes represent the nomogram-predicted probabilities grouped for each of the four quartile groups, along with the respective confidence intervals.

**Figure 4:**
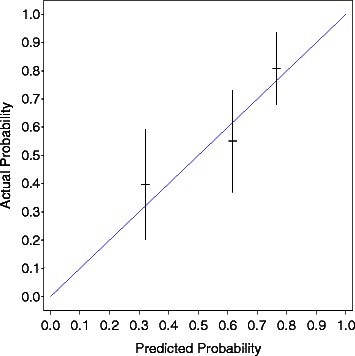
External calibration of nomogram for 3-year survival. The predicted survival rate from the nomogram was well correlated with the actual observation. The blue line indicates the ideal reference line where the predicted probabilities match the observed proportions. The dashes represent the nomogram-predicted probabilities grouped for each of the three tertile groups, along with the respective confidence intervals.

Internal and external validations were performed with the method described by Iasonos et al. [15]. Figure 3 shows the internal calibration of the nomogram for 5-year survival. The blue line indicates the ideal reference line at which predicted probabilities match the observed proportions. The dashes represent the nomogram-predicted probabilities grouped for each of the four quartile groups, along with the respective confidence intervals. The predicted survival rate from the nomogram was well correlated with the actual observation of 5-year survival in the training data. Figure 4 shows the external calibration of the nomogram for 3-year survival. The dashes represent the nomogram-predicted probabilities grouped for each of the three tertile groups, along with the respective confidence intervals. The predicted survival rate from the nomogram was also well correlated with the actual observation of 3-year survival in the validation data.

## Discussion

In this study, we developed a nomogram of OS of Japanese patients with bone-metastatic prostate cancer. This nomogram is an OS probability prediction tool comprising five pretreatment prognostic factors selected by multivariate analysis. These factors, namely patient age at initial treatment, pretreatment serum PSA level, clinical T stage, EOD, and biopsy Gleason sum, are common clinical factors and may be useful for all patients.

It is crucial to test the Cox model for proportionality of the hazards when building nomograms. We assessed the proportional hazards assumption by testing the interaction terms of the factors listed in Table 2 with time. Some violation of the proportional hazards assumption was evident (p = 0.012). However, if we include these interaction terms in the model, we cannot present the results as a simple nomogram because the effects of the covariates are time varying. Moreover, our nomogram was well calibrated in the internal and external data (Figures 3 and 4). Thus, we removed these interaction terms from the model. We also conducted multivariate analyses by treating age, log(PSA), and biopsy Gleason as trichotomous categorical covariates using tertiles as cut-off values. The estimated hazard ratios of each variable were at least monotonically increasing or decreasing over the categories, and thus we found no evidences that our chosen functional forms were inappropriate. Thus, we used these factors as continuous regressors.

Several groups have reported prognostic models for survival of patients with progressive disease. Almost all reports were of a prognostic nomogram of patients with CRPC; there are few reports on prognostic nomograms for hormone-naïve progressive prostate cancer before treatment [16-19].

Hussain et al. [15] reported various risk predictors of OS (SWOG9346). They identified baseline variables such as bone pain, performance status, Gleason sum, weight change, positive lymph node metastasis, pre-study PSA increments, and PSA level after treatment as strong prognostic factors for OS. Coopeberg et al. [13] also reported a large study on prostate cancer prognosis in hormone-naïve patients in Japan and the US. They assessed 13,740 US men and 19,265 Japanese men with prostate cancer and developed the Japan Cancer of the Prostate Risk Assessment (J-CAPRA). The CAPRA score, which ranges from 0 to 12 and is based on the Gleason sum, serum PSA level at initial treatment, and clinical stage, can predict progression-free survival after primary androgen deprivation therapy. Although the endpoint of the J-CAPRA is progression-free survival, our interest is in the development of an OS prognostic model for patients with hormone-naïve metastatic prostate cancer. Progression-free survival has been shown to be predictive of OS in men with CRPC [20], although the association between progression-free survival and OS is relatively weak. Some reports have indicated improvement in OS without an increase in progression-free survival [9] or improvement in progression-free survival without an increase in survival [21]. Accurate prediction models for prostate cancer survival probability would be valuable for patient counseling.

We analyzed the relationship between prostate cancer outcomes and pretreatment clinical factors and developed a prognostic nomogram of the OS of patients with bone metastasis. Recently, there has been rapid development in treatment for CRPC. In the US and other Western countries, some new effective agents for CRPC have been approved, such as docetaxel, cabazitaxel, sipuleucel-T, abiraterone, and enzalutamide [7-11,22]. Unfortunately, treatment for CRPC was still very limited in Japan until 2013 (cabazitaxel, abiraterone, and enzalutamide were approved in 2014), although docetaxel has been approved [12]. These agents could improve the survival of patients with CRPC.

External validation of this nomogram was performed using the validation data set of 102 cases. The predicted survival rate calculated by our nomogram was well correlated with practical observation. The docetaxel-use rate was significantly higher in the validation data set than in the training data set (p < 0.0001). Moreover, there were significant differences in incidence of clinical T4 disease between the training data and the validation data (p < 0.0001), although our nomogram was well correlated with actual observations.

As mentioned above, the first limitation of this study is that our nomogram was developed from data from the “pre-docetaxel era” or “docetaxel era.” For more accurate prediction for patients with prostate cancer in the “post-docetaxel era,” more recently collected data are needed. Moreover, validation samples collected from a non-Japanese population would provide wider applicability.

The second limitation of this study is the fact that patients enrolled in the study had various health statuses and complications. Our nomogram considers neither health status nor patient complications that may influence prostate cancer treatment outcomes [15,23,24]. Patients with prostate cancer are much older than those with other malignancies. Health status and complications should be classified in the rating score and included as predictive factors in the nomogram. Bone pain at diagnosis is also strong predictor of OS [15]. Unfortunately, data regarding pain at baseline were not available in this study.

The final limitation of this study is the lack of data about serum hemoglobin, lactate dehydrogenase, and alkaline phosphatase levels. These factors have been reported as predictive factors for patients with CRPC [18,19,25].

In conclusion, we developed a prognostic model for patients with bone-metastatic prostate cancer. This model could predict OS from five pretreatment factors, namely patient age at initial treatment, pretreatment serum PSA level, clinical T stage, EOD, and biopsy Gleason sum, in patients with bone-metastatic prostate cancer. External validation of this model showed it to be reasonably accurate and similar to practical actual survival probability.

## Conclusion

Our pretreatment prognostic nomogram might be useful for Japanese patients with bone-metastatic prostate cancer.

## Abbreviations

OS: Overall survival
EOD: Extent of disease on bone scan
CRPC: Castration-resistant prostate cancer
c-index: concordance index
PSA: Prostate-specific antigen
J-CAPRA: Japan Cancer of the Prostate Risk Assessment
